# Resignation and Appointment in the University of Maryland

**Published:** 1860-04

**Authors:** 


— *University of Maryland.*—Fifty young gentlemen received the Doctor's Diploma at the late Annual Commencement of this venerable school. The Chair of Anatomy and Physiology, vacated by the resignation of Prof. Joseph Roby, has been offered to Dr. William A. Hammond, Surgeon U. S. Army, who has accepted the offer, and will enter upon his duties with the commencement of the next session. Dr. Hammond is well known to our readers, as the author of the Essay on "*The Nutritive Value and Physiological Effects of Albumen, Starch and Gum, when singly and exclusively used as Food*," to which the prize was awarded at the Tenth Annual Meeting of the American

Medical Association. We wish him every success in a field of labor so congenial to his tastes.

